# Observation of Pain-Sensitive Points along the Meridians in Patients with Gastric Ulcer or Gastritis

**DOI:** 10.1155/2012/130802

**Published:** 2012-11-18

**Authors:** Hui Ben, Liang Li, Pei-Jing Rong, Zhi-Gao Jin, Jian-Liang Zhang, Yan-Hua Li, Xia Li

**Affiliations:** ^1^Institute of Acupuncture and Moxibustion, China Academy of Chinese Medical Sciences, Beijing 100700, China; ^2^Beijing Coal General Hospital, Beijing 100028, China; ^3^Beijing University of Traditional Chinese Medicine, Beijing 100029, China

## Abstract

This study aims to investigate the sensitization of human skin points along certain meridians related to visceral disease by using the pressure-pain threshold (PPT) as an indicator. We detected and compared the PPTs of people with and without gastric ulcer or gastritis on the related acupoints, abdomen area, and back area with von Frey detector and observed the similarities and differences under their respective physiological and pathological states. The results showed that (1) the PPTs of patients with gastric ulcer on related acupoints decreased significantly compared with the control group; (2) there was no significant difference in PPT between the chosen points of the measured meridian and the adjacent nonacupoints; (3) there was an apparent distribution of tender points on the relevant abdomen and back regions of patients with gastric ulcer or gastritis, but none was found on the control group; (4) the pain-sensitive points of gastric ulcer and gastritis patients were BURONG (ST19), LIANGMEN (ST21), and HUAROUMEN (ST24) of the stomach meridian on the abdominal region and PISHU (BL20), WEISHU (BL21), and WEICANG (BL50) on the back, among others The results suggest that the practical significance of acupoints may lie in its role as a relatively sensitive functional area. In a pathological state, the reflex points on the skin which are related to certain visceral organs become sensitive and functionally intensify.

## 1. Introduction

The pressure-pain threshold (PPT) of skin is the minimum force applied on the skin by external pressure which induces pain. It is one of the traditional measures to quantitatively evaluate pain that has been widely used in basic as well as clinic studies. In most cases, reporting of perceived pain by the subjects is usually influenced by factors like the expectancy, and pressure algometry however provides objective information of the local pain conditions.

According to the theory of meridians in traditional Chinese medicine, there are connections between the internal organs and their respective somatic meridians. Therefore, visceral lesions can lead to changes of pain sensation, which exhibited as tenderness point(s), or pigmentations and so forth in certain areas of the body surface. And similarly, the stimulation of acupoints can result in the regulation of function of the responding internal organs.

Previous studies have shown that the sensitive points on the body surface accompanied with some visceral disorders including the digestive diseases which are characterized with abdominal pain and tenderness as the common sign and symptom. In this study, by the help of von Frey detector, we measured the PPT of acupoints along meridians of subjects with gastric ulcer or gastritis to assess their sensitization under the pathological conditions.

## 2. Materials and Methods

### 2.1. Detection of Pressure-Pain Threshold

#### 2.1.1. Detection Equipment

The 2390-type von Frey detector produced by the IITC Company of the United States was used to detect the PPTs of acupoints along the related meridians so as to compare the similarities and differences between the results acquired in normal and pathological states.

#### 2.1.2. Detection of Acupoints

*Tested acupoints* (1) Stomach Meridian: ZUSANLI (ST36), SHANGJUXU (ST37), XIAJUXU (ST39); (2) Large Intestine Meridian: SHOUWULI (LI13), QUCHI (LI11), SHOUSANLI (LI10), HEGU (LI4); (3) back acupoints: WEISHU (BL21), DACHANGSHU (BL25); (4) points which are 1.0–1.5 cm adjacent to the above acupoints were selected as the control acupoints; (5) the abdominal and back acupoints.

#### 2.1.3. Methods

The probe tip was kept moving vertically downward toward the skin at an even speed. When the subject felt the pain, the probe was removed immediately and the data on the detector were recorded simultaneously. Each point was tested three times at an interval of three minutes. The average of the data was taken as the threshold.

### 2.2. Partition of the Testing Zones on the Abdomen and Back

**Figure 1:**
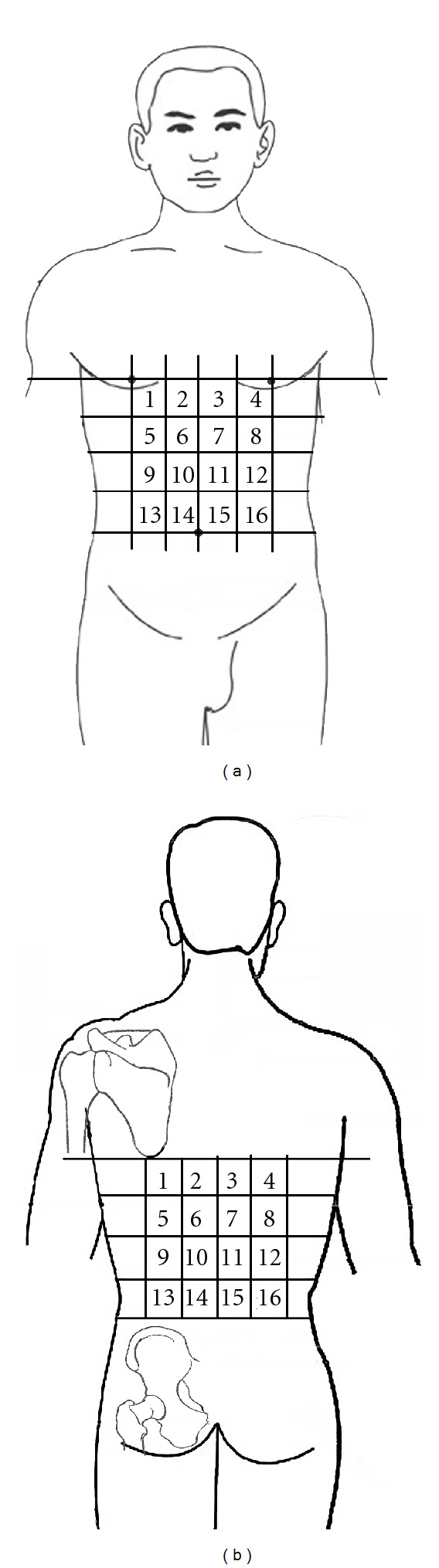
Abdominal and back testing zones. The abdominal and back regions of the subject were divided into 16 testing zones, respectively.

The abdominal and back regions of the subject were divided into 16 testing areas, respectively (Figure 1). In each testing area three points were tested, and each point had a certain distance to the other two points.

### 2.3. Identification of Tender Points on the Abdomen and Back

Tender points in the testing zones of the subject's abdomen and back were identified by finger-pressure method. Tender points were determined when the subject expressed obvious pain or a quasistringy pain while the thumb pressed vertically downward at an even speed.

### 2.4. Selection of Pain-Sensitive Points on the Abdomen and Back

Data are expressed as means of the values acquired from the three test points in each of the 16 testing zones mentioned above. The three points with the lowest pain threshold (i.e., pain-sensitive point) were selected and marked in Figure 5. The points marked for each group were cumulatively displayed in Figure 5 to demonstrate the different responses to pain of the relevant areas on the surface of body between healthy people and patients with gastric ulcer or gastritis.

### 2.5. Grouping

#### 2.5.1. Control Group 1

Twenty healthy volunteers were selected (10 male and 10 female, aged 25–50). The PPTs were measured, respectively, on the stomach meridian, large intestine meridian, back meridian, and those adjacent to open acupoints.

#### 2.5.2. Gastric Ulcer Group 1

Sixteen volunteers with gastric ulcer were selected (8 male and 8 female, aged 30–55). The PPTs were measured, respectively, on the stomach meridian, large intestine meridian, back meridian, and those adjacent to open acupoints.

#### 2.5.3. Control Group 2

Twenty one healthy volunteers were selected (11 male and 10 female, aged 30–55). The PPTs were measured, respectively, in each testing zone on the abdomen and back.

#### 2.5.4. Gastric Ulcer Group 2

Sixteen volunteers with gastric ulcer were selected (8 male and 8 female, aged 30–55). The PPTs were measured, respectively, in each testing zone on the abdomen and back.

#### 2.5.5. Gastritis Group

Twenty one volunteers with gastritis were selected (9 male and 12 female, aged 30–60). The PPTs were measured, respectively, in each testing zone on the abdomen and back.

## 3. Results

### 3.1. The Comparison between Gastric Ulcer Group 1 and Control Group 1

#### 3.1.1. The Comparison of PPTs between the Gastric Ulcer Group and the Control Group

**Figure 2:**
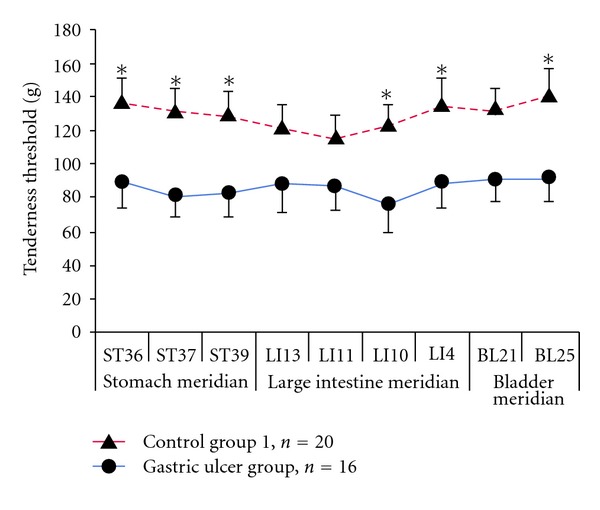
The PPTs of the gastric ulcer group decreased significantly compared with that of the control group.

The results showed a significant decrease of PPTs of the test points on related meridians of patients with gastric ulcer in disease state compared with the control group (*P* < 0.05) (Figure 2).

#### 3.1.2. The Comparison of PPTs between the Selected Acupoints and Adjacent Points

**Figure 3:**
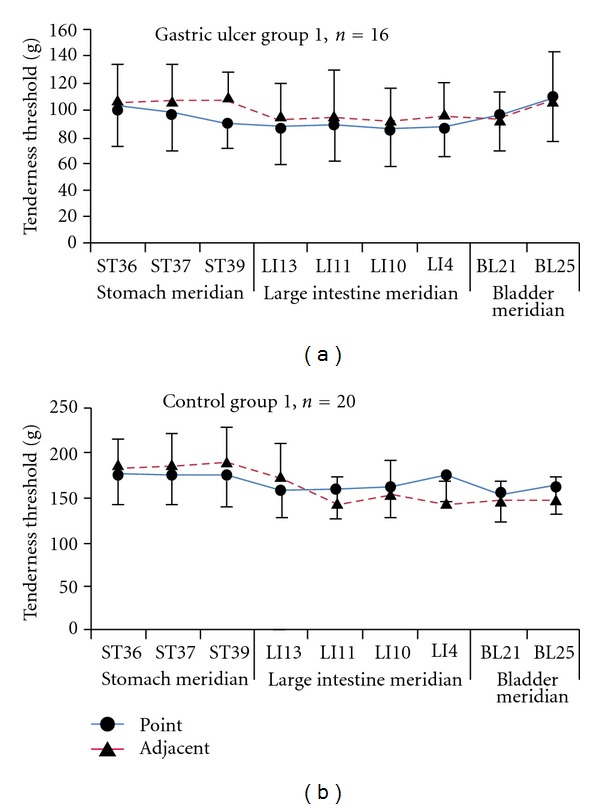
There was no significant difference in PPTs between points on meridians and nonmeridians.

There was no significant difference in PPTs between the points along the tested meridians and the adjacent open points (Figure 3).

### 3.2. The Comparison of PPTs among Gastric Ulcer Group 2, Gastritis Group, and Control Group 2

#### 3.2.1. The Distribution of Tender Points on the Abdomen and Back

**Figure 4:**
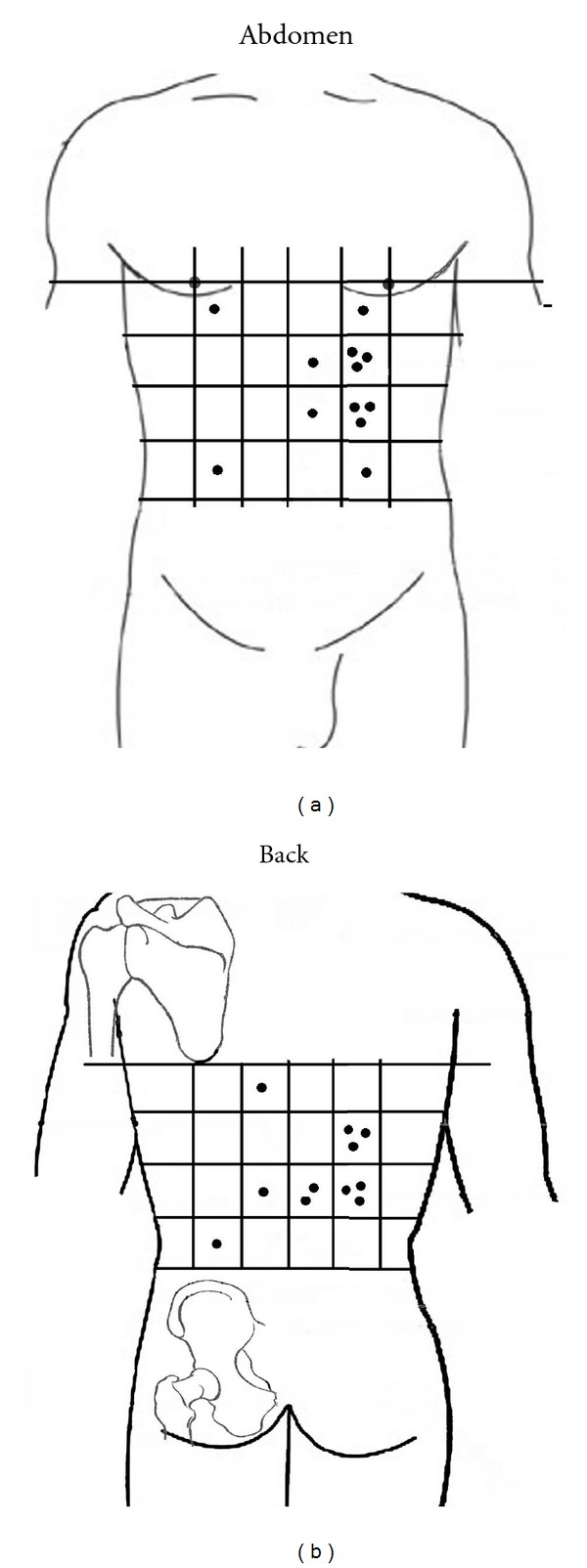
There were more tender points distributed on the left side of the abdomen and the lower right side of the back in the gastric ulcer group.

The distribution of tender points in the abdominal and back testing zones was detected by using the finger-pressure method. The results showed that there was no apparent tender point in the normal control group, but, in the gastric ulcer group, tender points appeared more often on the left side of the abdomen and the lower right side of the back (Figure 4).

#### 3.2.2. The Distribution of the Pain-Sensitive Points on the Abdomen and Back

**Figure 5:**
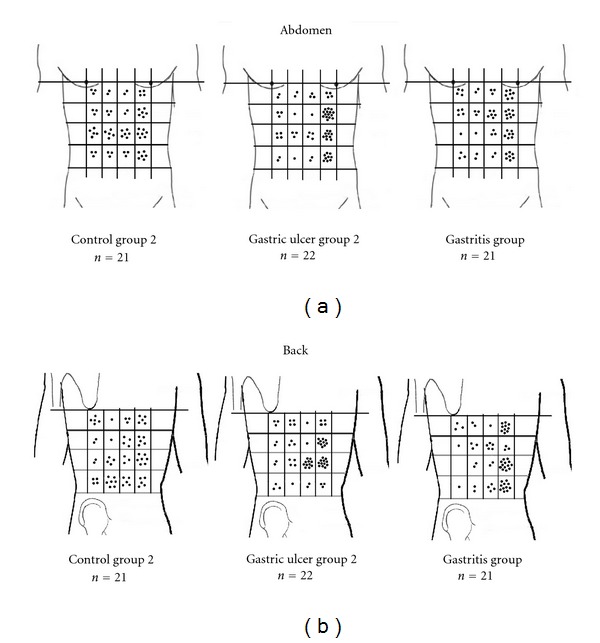
(a) The distribution of the pain-sensitive points on the abdomen. (b) The distribution of the pain-sensitive points on the back.

**Figure 6:**
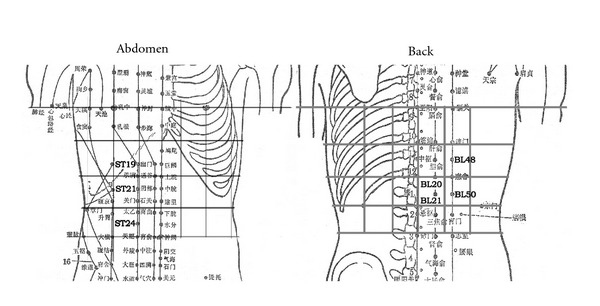
The pain-sensitive points were mainly distributed at ST19, ST21, ST24, among others of the stomach meridian on the abdomen and at BL20, BL21, BL48, BL50, among others of the bladder meridian on the back.

The PPTs of pain-sensitive points displayed in Figures 5(a) and 5(b) were measured by von Frey detector. Figure 5 shows a dispersed distribution of pain-sensitive points on the abdomen and back in the control group, and there was no relative specificity. The pain-sensitive points of the gastric ulcer group were relatively concentrated. They were mainly distributed at BURONG (ST19); LIANGMEN (ST21), and HUAROUMEN (ST24), among others of the stomach meridian on the abdomen and PISHU (BL20), WEISHU (BL21), YANGGANG (BL48), WEICANG (BL50), among others of the bladder meridian on the back (Figure 6). The distribution of the pain-sensitive points was basically consistent with the distribution of tender points obtained by using the finger-pressure method. In the gastritis group, the distribution of the pain-sensitive points was less concentrated than that of the gastric ulcer group. It may be attributed to the fact that the clinical symptoms of patients with gastritis were less severe than that of patients with gastric ulcer.

## 4. Discussion

Mechanical tenderness is not only a symptom of local skin or muscle tissue, but also a typical performance of some pain syndromes. It has diagnostic significance in clinical settings. The PPT can be used as an indicator for the evaluation of the body's inflammatory activity, subcutaneous tissue sensitivity, and pain tolerance. It has been applied in the assessment of the efficacy of various clinical treatment therapies [1, 2].

Research has shown that the primary afferent transmission of pain may be affected by the neuroendocrine interaction [3]. As visceral diseases increase the chemical substances that cause pain (5–HT, histamine, peptides, and others) in the body, the pain sensitivity of the patients increases as well [4]. In 1999, British scholar Kosek [5] reported that the PPTs at the muscle-nerve points were significantly lower than the PPTs of “pure” muscle and/or bone. These “muscle-nerve” points are similar to what we call the meridian points.

Meridian points are the reaction points and the treatment points of visceral diseases in traditional Chinese Medicine. The correlation between meridians and internal organs means that the meridians not only have close correlation with their corresponding organs in terms of physiological function, but also demonstrate specific reactions of visceral disorders on the surface of body in pathological states. The therapeutic stimulation on meridians has a regulatory effect on the function of internal organs [6]. In recent years, Yu et al. have proposed the idea that acupoints are “dynamic” and the functioning of acupoints is a dynamic process [7]. He believes that area size of acupoints on the body surface and the function of acupoints are not fixed. On the contrary, the function and area size of acupoints change with the state of the body and the function of their corresponding internal organs. He suggests that acupoints have two states, namely, on/off. The switch between the two states is a dynamic process and reflects the transition of the body from a “dormant” healthy state to an “activated” pathological state. At the same time, the process is accompanied with changes of the microphysical and chemical environments; that is, at the time of visceral disorder, the acupoints turn from the dormant state to the sensitized state. Thus it is possible to regulate the functioning of internal organs by stimulating acupoints. 

In recent years, many studies on acupoint specificity have shown that the function and characteristics of acupoints are closely related to the distribution of specific nerves [8, 9] and blood vessels [10, 11]. Acupoints also exhibit unique physical properties, such as optical specificity [12], thermal specificity [13], and electromagnetic specificity [14]. In addition, gene expression in the brain caused by stimulating acupoints has significantly increased comparing with the gene expression caused by stimulating nonacupoints. The result suggests that the effect of acupuncture on acupoints is different from the effect of acupuncture on nonacupoints [15]. Another study has shown that acupuncture can regulate the nitric oxide (NO) content and the activity of nitric oxide synthase (NOS) in body tissues [16–18]. The increase of NO content in the pathological state reflects the activity of the acupoints from one side.

Results showed that the PPTs of skin in the gastric ulcer group were significantly lower than that of the normal control group, regardless of whether it was tested on related meridians or on the adjacent open points. There was no significant difference in PPTs between points on the meridians and adjacent open points. It showed that in pathological states the meridian points were more sensitive and had lower PPTs than in the normal state. Meridian points had no significant difference compared with the adjacent open points. It suggests that acupoint was not just an isolated point but represents a relatively sensitive point area with certain special function. Previous studies have shown that meridian has an optimized and inclusive systematic structure instead of a single channel structure [19].

The distribution of pain-sensitive points on the abdomen and back also suggests that, in the gastric ulcer group, there were obvious tender points in the reflex zones on the body surface associated with the disease. The PPTs were lower, and the pain-sensitive points were more concentrated. Tender points and pain-sensitive points were mainly concentrated at ST19, ST21, and ST24 on the Stomach Meridian of Foot-Yangming of the abdomen and at BL20, BL21, BL48, and BL50 on the Bladder Meridian of Foot-Taiyang of the back. A previous research showed that the LIANGMEN point area on the abdomen and the WEISHU point area on the back had a clear overlap with the segmental distribution of nerves of the stomach [20]. The Foot-Yangming Meridian points and the back points were closely related to the stomach [21, 22]. According to the responses of subjects, they felt apparent pain or tingling when these areas were pressed or tested, and sometimes the string pain goes up and down along the meridian. It is evident that there is a relatively specific link between the meridians (acupoints) on the body surface and the stomach diseases. But based on the feeling of subjects and the tested results, the correlation was not apparent in all states. We observed that there was no tender point on the body of subjects in the normal control group, and the distribution of pain-sensitive points was relatively dispersed. But in pathological states the subjects felt clear pain at meridian points (acupoints). This showed that, in pathological states, acupoints and meridian areas on the body surface were more sensitive and active compared with the normal state. This performance was positively correlated to the alleviation of clinical symptoms and pain [1, 2].

The above results suggest that the practical significance of acupoint may lie in its role as a relatively sensitive functional area. The reflex area of internal organs on the surface of body is more sensitive and functionally more active than other areas of the body surface, particularly in pathological states. Therefore, acupoints (point areas) cannot only “mirror the disease” in diagnosis, but also help to “cure the disease” in treatment.

## 5. Conclusions

In conclusion, tender points appeared on the abdomen and back regions of patients with gastric ulcer or gastritis, suggesting that the essence of acupoints goes beyond a mere site for stimulation. Thus, our study provides scientific evidence for the theory of “correlation between meridians and viscera” and, further, helps elucidate the mechanism of acupuncture in the management of gastrointestinal diseases.

## Appendix

The positions of mentioned acupoints in this paper

**ST19: **on the upper abdomen, 6 *cun* above the centre of the umbilicus and 2 *cun* lateral to the anterior midline.
**ST21:** on the upper abdomen, 4 *cun* above the centre of the umbilicus and 2 *cun* lateral to the anterior midline.
**ST24:** on the upper abdomen, 1 *cun* above the centre of the umbilicus and 2 *cun* lateral to the anterior midline.
**ST36:** on the anteriolateral side of the leg, 3 *cun* below Dubi (ST35), one finger breadth (middle finger) from the anterior crest of the tibia.
**ST37: **on the anteriolateral side of the leg, 6 *cun* below Dubi (ST35), one finger breadth (middle finger) from the anterior crest of the tibia.
**ST39: **on the anteriolateral side of the leg, 9 *cun* below Dubi (ST35), one finger breadth (middle finger) from the anterior crest of the tibia.
**LI10: **2 *cun *above Quchi (LI11) when a fist is made.
**LI11: **between the humerus and radius when the elbow is flexed and the hand is put on the chest.
**LI13:** 3 *cun* above the elbow, mid the Zhouliao (LI12), and on the big vessel (probably the cephalic vein), when the elbow is flexed.
**LI14: **7 *cun *above the elbow, at the lower end of the deltoid muscle.
**BL20:** on the back below the spinous process of the 11th thoracic vertebra, 1.5* cun* lateral to the posterior midline.
**BL21:** on the back below the spinous process of the 12th thoracic vertebra, 1.5* cun* lateral to the posterior midline.
**BL25:** on the low back, below the spinous process of the 4th lumbar vertebra, 1.5* cun *lateral to the posterior midline.
**BL48:** on the back, below the spinous process of the 10th thoracic vertebra, 3* cun *lateral to the posterior midline.
**BL50:** on the back, below the spinous process of the 12th thoracic vertebra, 3* cun *lateral to the posterior midline.
